# Curcumin Protects Neuron against Cerebral Ischemia-Induced Inflammation through Improving PPAR-Gamma Function

**DOI:** 10.1155/2013/470975

**Published:** 2013-05-16

**Authors:** Zun-Jing Liu, Wei Liu, Lei Liu, Cheng Xiao, Yu Wang, Jing-Song Jiao

**Affiliations:** ^1^Department of Neurology, China-Japan Friendship Hospital, 2 Yinghua Dongjie, Hepingli, Beijing 100029, China; ^2^Clinical Research Institute, China-Japan Friendship Hospital, 2 Yinghua Dongjie, Hepingli, Beijing 100029, China

## Abstract

Cerebral ischemia is the most common cerebrovascular disease worldwide. Recent studies have demonstrated that curcumin had beneficial effect to attenuate cerebral ischemic injury. However, it is unclear how curcumin protects against cerebral ischemic injury. In the present study, using rat middle cerebral artery occlusion model, we found that curcumin was a potent PPAR**γ** agonist in that it upregulated PPAR**γ** expression and PPAR**γ**-PPRE binding activity. Administration of curcumin markedly decreased the infarct volume, improved neurological deficits, and reduced neuronal damage of rats. In addition, curcumin suppressed neuroinflammatory response by decreasing inflammatory mediators, such as IL-1**β**, TNF-**α**, PGE2, NO, COX-2, and iNOS induced by cerebral ischemia of rats. Furthermore, curcumin suppressed I**κ**B degradation that was caused by cerebral ischemia. The present data also showed that PPAR**γ** interacted with NF-**κ**B-p65 and thus inhibited NF-**κ**B activation. All the above protective effects of curcumin on cerebral ischemic injury were markedly attenuated by GW9662, an inhibitor of PPAR**γ**. Our results as described above suggested that PPAR**γ** induced by curcumin may play a critical role in protecting against brain injury through suppression of inflammatory response. It also highlights the potential of curcumin as a therapeutic agent against cerebral ischemia.

## 1. Introduction

Cerebral ischemia is the most common cerebrovascular disease, and it is one of the leading causes of morbidity and mortality worldwide. A transient or permanent local reduction of cerebral blood flow causes cerebral ischemia with a condition of complex pathology. Excitatory amino acid toxicity, oxidative stress, intracellular calcium overload, inflammation, and apoptosis are involved in the pathological process after cerebral ischemic injury [1]. Among these pathological changes, inflammatory response is the most important, which is mediated by nuclear factor kappa B (NF-*κ*B) signal transduction pathway. Activation of NF-*κ*B promotes proinflammatory cytokines and enzymes including tumor necrosis factor *α* (TNF-*α*), interleukins (ILs), nitric oxide (NO), prostaglandin E2 (PGE2), cyclooxygenase-2 (COX-2), and inducible nitric oxide synthase (iNOS), which may ultimately induce neuronal damage [2]. 

The peroxisome proliferator-activated receptors (PPARs), including *α*, *γ*, and *δ*/*β*, encoded by separate genes, are members of the nuclear receptor superfamily of ligand-activated transcription factors [3], among which PPAR*γ* has been the most characterized, in part, due to its therapeutic potential for diabetes and related consequences such as metabolic syndrome. PPAR*γ* is predominantly expressed in adipose tissue, immune system [4], and central nervous system [5, 6]. Normally, PPAR*γ* regulates gene transcription by binding to conserved DNA sequences termed *peroxisome proliferator response elements* (*PPRE*). Studies have shown that activation of PPAR*γ* contributed to neuroprotection in AD [7], PD [8], and cerebral ischemia [9, 10]. The beneficial effects of PPAR*γ* on neurons were due to the suppression of inflammatory response [11] via inhibiting the activation of NF-*κ*B pathway [12]. 

The poor prognosis of cerebral ischemia is largely due to the lack of effective therapies. Even though a large number of compounds have been proven to reduce cerebral ischemic injury, clinical trials have been unsuccessful because of toxic side effects. Thus, the development of new drugs and the discovery of novel mechanisms for treating cerebral ischemia are urgently needed. Due to the crucial role of inflammation in the progression of ischemic brain injury, a search for novel agents targeting inhibition of inflammatory response after cerebral ischemia has been initiated [13]. Curcumin is a yellow colored phenolic pigment obtained from powdered rhizome of *C. longa Linn*. Studies have shown that curcumin has multiple pharmacological actions, such as antioxidant [14], anti-inflammatory [15], and anticancer [16] properties. Recent studies have demonstrated that curcumin protected neurons from cerebral ischemic injury [17, 18]. In addition, our previous study showed that curcumin was an agonist of PPAR*γ* in U937 cells [19]. Owing to the important role of PPAR*γ* on cerebral ischemia, we speculated whether curcumin protected against cerebral ischemic injury through activating PPAR*γ* signaling.

Middle cerebral artery occlusion (MCAO) in rat is widely used to study experimental ischemic injury and has provided invaluable understanding of the pathophysiology of focal cerebral ischemia. In the present study, using rat MCAO-induced cerebral ischemia model, we found that curcumin markedly decreased the infarction volume, improved neurological deficits, and reduced neuronal damage. The beneficial effects of curcumin on cerebral ischemia might be due to its activating PPAR*γ* pathway, and ultimately suppressed neuroinflammatory response.

## 2. Materials and Methods

### 2.1. Reagents

Curcumin, GW9662, MG132, 2,3,5-triphenyltetrazolium chloride (TTC), and Griess reagent were purchased from Sigma. Rat IL-1*β*, TNF-*α*, and PGE2 ELISA kits were products of R&D company. PPRE and NF-*κ*B consensus oligonucleotides labeled with biotin were synthesized by the IDT Company. Electrophoretic mobility shift assay (EMSA) kit and coimmunoprecipitation (Co-IP) kit were purchased from Pierce. Rabbit anti-NF-*κ*B p65 and rabbit anti-I*κ*B*α* polyclonal antibodies were purchased from Santa Cruz Biotechnology. Rabbit anti-COX-2, rabbit anti-PPAR*γ*, and rabbit anti-iNOS polyclonal antibodies were purchased from Millipore.

### 2.2. Animals and Treatments

Sprague-Dawley male rats (280–310 g) were supplied by Beijing Vital River Animal Center. They were housed four per cage in a standard animal room with a 12 h light/dark cycle and with free access to food and water. NIH Guidelines for the Care and Use of Laboratory Animals were followed in all animal experimental procedures. The rats were randomly divided into sham-operated group, MCAO group, curcumin 200 mg/kg + MCAO group, curcumin 200 mg/kg + GW9662 4 mg/kg + MCAO group, MG132 + MCAO group, and MG132 alone group, and the number of rats in each group was 20.

Curcumin 200 mg/kg and PPAR*γ* inhibitor GW9662 4 mg/kg were dissolved in 10% dimethyl sulfoxide (DMSO), and they were intraperitoneally (i.p., 1 mL/kg) injected to rats for continuous 3 days. One hour after the last injections of curcumin and GW9662, the rats were subjected to MCAO for 2 h and reperfused for 24 h. Sham-operated rats received 10% DMSO only. In the case of NF-*κ*B inhibition, 2 mg/kg of MG132 (Z-Leu-Leu-Leu-aldehyde) dissolved in DMSO was injected intraperitoneally (i.p., 0.5 mL/kg) to sham-operated (MG132 alone group) or MCAO (MG132 + MCAO group) rats for continuous 3 days, respectively.

### 2.3. MCAO

Under chloral hydrate anesthesia (350 mg/kg, intraperitoneally), a 4/0 surgical nylon monofilament tip flattened by sandpaper and coated with 0.01% poly-L-lysine was introduced into the left internal carotid artery through the external carotid stump, advanced 18–20 mm after the carotid bifurcation until a slight resistance was felt. Such resistance indicated that the filament had passed beyond the proximal segment of the anterior cerebral artery. At this point, the intraluminal filament blocked the origin of the middle cerebral artery and occluded all sources of blood flow from the internal carotid artery, anterior cerebral artery, and posterior cerebral artery. Throughout the procedure, body temperature was maintained at 37 ± 0.5°C with a thermostatically controlled infrared lamp. The filament was left in place for 2 h and then withdrawn. Animals were then returned to their cages and closely monitored until they recovered from anesthesia. The sham-operated rats were treated identically, except that the middle cerebral arteries were not occluded after the neck incision. Rats were subjected to MCAO for 2 h and reperfused for 24 h.

### 2.4. TTC Staining

After 24 of reperfusion, the rats were deeply anesthetized with chloral hydrate and then decapitated, after which the brains were rapidly removed. The brains (*n* = 6 in each group) were sliced into 2 mm thick coronal sections and stained with standard 2% TTC for 10 min at 37°C followed by overnight immersion in 10% formalin. The infarct zone was demarcated and analyzed by the Mias-2000 image analysis system (Institute of Image and Graphics, Sichuan University, China). Infarct areas of all sections were added to derive the total infarct area, which was multiplied by the thickness of the brain sections to obtain the infarct volume. The infarct volume was evaluated by Image-Pro Plus 5.1 analysis system (Media Cybernetics Inc., USA) using Swanson's method which corrects for edema [20].

### 2.5. Neurological Behavioral Test

Neurological deficits of rats (*n* = 10 in each group) were measured after 24 h of reperfusion according to the method of Longa et al. [21]. Neurological deficits were scored on a 5-point scale. Zero indicated that, rats extended both forelimbs towards the floor when gently suspended 1 m above the floor and with no other signs of neurological deficit; 1 indicated that rats consistently flexed the forelimb contralateral to MCAO; 2 indicated that rats circled towards the contralateral side when the tail was pulled; 3 indicated that rats spontaneously circled towards the contralateral side when allowed to move freely; 4 indicated no spontaneous movement with an apparent depressed level of consciousness. The neurobehavioral test was performed by an investigator who was blinded to the experiment. 

### 2.6. Nissl Staining

Five rats were taken from each group for quantitative Nissl staining. The brain sections were Nissl-stained with toluidine blue for neuronal cell bodies. The brain sections were then mounted, air-dried, dehydrated, and cover slipped. One in every four sections was taken from a continuous series of sections prepared from cortex. Six sections were selected in each mouse, and the number of positively stained cells in each group was counted. The mean of the number of positively stained cells was calculated from 30 sections of each group. The prepared sections were observed by an investigator who was blinded to the experiment using light microscopy (NIKON E600, Japan). Images were analyzed using the Image-Pro plus system. 

### 2.7. Western Blot Analysis

Rat cortex was homogenized in nondenaturing lyses buffer. The 30 *μ*g sample proteins were separated by SDS-polyacrylamide gel electrophoresis (SDS-PAGE) in a 10% polyacrylamide gel and transferred to a polyvinylidene difluoride (PVDF) membrane. The membranes were blocked in 5% skim milk-TBS-T (20 mM Tris-HCl, pH 7.5, 500 mM NaCl. 0.1% Tween 20) at 4°C overnight. The blots were probed with antibodies against NF-*κ*B p65, I*κ*B*α*, COX-2, PPAR*γ*, and iNOS in 5% skim milk-TBS-T for 2 hours at room temperature. After washing, the blot was then incubated with horseradish peroxidase-conjugated secondary antibody in skim milk-TBST for 2 hours at room temperature. The blot was developed with LAS3000 chemiluminescence system (Fujifilm, Tokyo, Japan) and the densities of the bands were determined using Gel-Pro Analyzer 4.0 software.

### 2.8. Electrophoretic Mobility Shift Assay (EMSA)

 Rat cortex nuclear extracts for EMSA were prepared using nuclear-cytosol extraction kit. Annealed double-stranded PPRE oligonucleotides (5′-CGT CAT CCC AGG GCA AAG TAC AAA GAG CCA GG-3′) and NF-*κ*B consensus oligonucleotides (5′-GCC TGG GAA AGT CCC CTC AAC T-3′) labeled with biotin were synthesized by IDT. EMSA kit was used to perform the reaction. The binding reaction (20 *μ*L in total) consists of 10 *μ*g of protein extracts, 20 fmol of biotin labeled DNA, 2.5% glycerol, 5 mM MgCl_2_, and 50 ng/*μ*L poly (dI·dC) and incubated for 20 min at room temperature. DNA-protein complexes were resolved by electrophoresis on a 6% polyacrylamide gel at 4°C in 0.5x TBE buffer (45 mM Tris borate, 1 mM EDTA) and transferred to a nylon membrane. Then the membrane was detected with the enhanced LAS3000 chemiluminescence system.

### 2.9. Coimmunoprecipitation (Co-IP)

Rat cortex was lysed in nondenaturing lysis buffer. The Co-IP assay was performed following the protocol of Co-IP kit. Briefly, 50 *μ*g of the purified PPAR*γ* antibody was immobilized in 100 *μ*L, 50% antibody coupling gel; 300 *μ*g protein extracts were incubated with gentle end-over-end mixing for 2 hours at room temperature. Immunoprecipitated complexes were eluted thrice with 50 *μ*L elution buffer, boiled and separated by SDS-PAGE, transferred to a PVDF membrane, incubated with NF-*κ*B antibody, and detected with the enhanced LAS3000 chemiluminescence system.

### 2.10. Cytokine Assay by ELISA

Rats were decapitated and the cortex tissues were isolated. Then they were dissected, snap-frozen on dry ice, homogenized, and diluted in the provided buffer containing protease inhibitor tease. IL-1*β*, TNF-*α*, Cox-2, and PGE2 were measured by sandwich ELISA kits following the manufacturer's instructions. Determinations were performed in duplicate and the results were expressed as pg/mL.

### 2.11. NO Assay

Rat cortex was homogenized and measured for the accumulation of nitrite (NO^2−^), a stable breakdown product of NO with the Griess reagent. Briefly, Griess reagent was added to an equal volume of supernatant (100 *μ*L) and incubated for 20 minutes at room temperature. The optical density was measured at 540 nm and a standard curve established using NO^2−^ at a range of 1 to 100 *μ*M. The amount of NO in the sample was calculated using a sodium nitrite standard curve freshly prepared in culture medium.

### 2.12. Statistical Analysis

The SPSS 13.0 computer program was used for all calculations and statistical evaluations. Differences among different groups were tested by one-way ANOVA with LSD test. Results were presented as means ± SD and a level of *P* < 0.05 was considered as significant.

## 3. Results

### 3.1. Curcumin Induced PPAR*γ* Expression and Activation in Cerebral Ischemia of Rats

**Figure 1:**
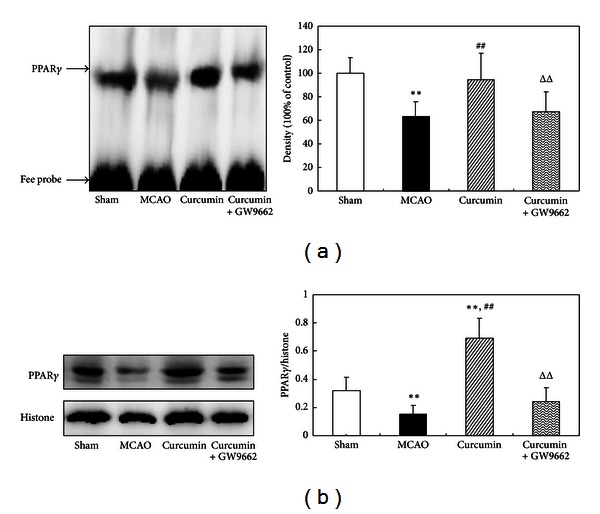
Curcumin induced PPAR*γ* expression and activation in cerebral ischemia of rats. Rats were injected (i.p.) with curcumin and GW9662 for continuous 3 days. One hour after the last injections, the rats were subjected to MCAO for 2 h and reperfused for 24 h. Rat cortex was isolated for detection of PPAR*γ*-PPRE binding activity and PPAR*γ* expression. (a) EMSA assay of PPAR*γ*-PPRE binding activity. (b) Western blot assay of PPAR*γ* expression. Data were expressed as mean ± SD. The EMSA and western blot images were representative of four independent experiments demonstrating similar results. **P < 0.01 versus sham-operated rats, ^##^ *P* < 0.01 versus MCAO rats, and ^ΔΔ^ *P* < 0.01 versus curcumin-treated rats.

In this study, we examined the effects of curcumin on PPAR*γ* expression and activity in a rat model of cerebral ischemia. Our results showed that the expression of PPAR*γ* significantly increased after curcumin treatment. And a promoted PPAR*γ*-PPRE binding activity was also observed in rat cortex. The overexpression and activation of PPAR*γ* induced by curcumin were counteracted by coadministration of GW9662, an inhibitor of PPAR*γ* (Figure 1), suggesting that curcumin was a potent agent to promote PPAR*γ* activity.

### 3.2. Curcumin Reduced Infarct Volume and Improved Neurobehavioral in Cerebral Ischemia of Rats

**Figure 2:**
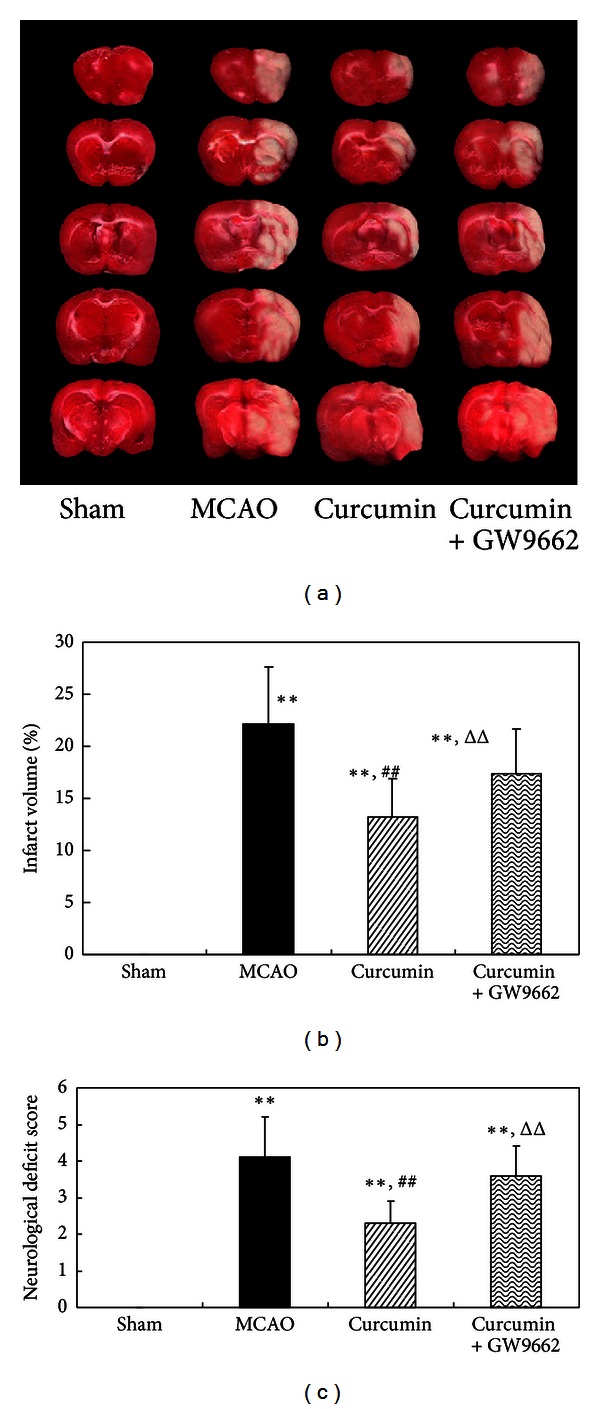
Curcumin reduced infarct volume and improved neurological behavior in cerebral ischemia of rats. Rats were injected (i.p.) with curcumin and GW9662 for continuous 3 days. One hour after the last injections, the rats were subjected to MCAO for 2 h and reperfused for 24 h. (a) TTC staining of the brain. (b) Infarct volume of cerebral cortical and subcortical areas. Data were expressed as mean ± SD with 6 individual rats per group. (c) Determination of neurological deficits. Data were expressed as mean ± SD with 10 individual rats per group. **P < 0.01 versus sham-operated rats, ^##^ *P* < 0.01 versus MCAO rats, and ^Δ^ *P* < 0.05 versus curcumin-treated rats.

The above results showed that curcumin can elevate PPAR*γ* activity in rat model of cerebral of ischemia. We next investigated whether the activated PPAR*γ* induced by curcumin contributed to neuroprotection. Rats were subjected to MCAO for 2 h and reperfused for 24 h, and the body temperature of rat was not changed during the experiment (data not shown). The extensive infarction was detected in the cerebral cortical and subcortical areas over a series of brain sections by TTC staining. As a result, curcumin significantly reduced infarct volume in comparison with sham-operated rats. Coadministration of GW9662 markedly attenuated the effect of curcumin on infarct volume of rat (Figures 2(a) and 2(b)). 

We next investigated curcumin on neurological deficits. No neurological deficit was observed in sham-operated rats. Rats exhibited focal neurological deficits following MCAO with failure to fully extend the forepaw. Treatment with curcumin showed a significant decrease in neurological score, indicating that curcumin can improve neurological behavior. Coadministration of GW9662 significantly attenuated the ameliorative effects of curcumin on neurological behavior (Figure 2(c)). These results suggested that the neuroprotection of curcumin against cerebral ischemic injury was associated with the activation of PPAR*γ*.

### 3.3. Curcumin Reduced Neuronal Injury in Cerebral Ischemia of Rats through Activating of PPAR*γ*

**Figure 3:**
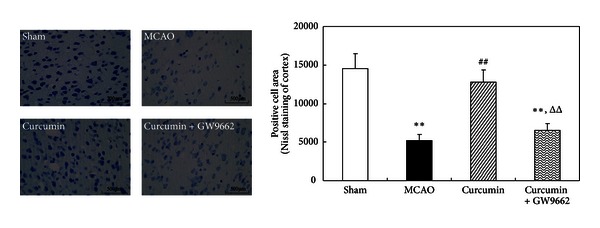
Curcumin reduced neuronal injury in cortex of cerebral ischemic rat. Rats were injected (i.p.) with curcumin and GW9662 for continuous 3 days. One hour after the last injections, the rats were subjected to MCAO for 2 h and reperfused for 24 h. Rats were sacrificed for Nissl staining. Data were expressed as mean ± SD with 5 individual rats per group. **P < 0.01 versus sham-operated rats, ^##^ *P* < 0.01 versus MCAO rats, and ^Δ^ *P* < 0.05 versus curcumin-treated rats.

To correlate behavioral changes with histochemical modifications in the brain of rats, Nissl staining of neuronal cell was performed. The number of Nissl-positive cells reduced greatly in cortex of rats subjected to MCAO compared with sham-operated rats. Curcumin significantly increased the number of Nissl-positive cells in rat cortex (Figure 3), while coadministration of GW9662 significantly attenuated the inhibitory effects of curcumin on neuronal damage. The morphological change induced by curcumin was a clear indication of its neuroprotective effects. These results verified that curcumin attenuated the neuronal injury and dysfunction through activating PPAR*γ*.

### 3.4. Curcumin Inhibited Neuroinflammatory Response in Cerebral Ischemia of Rat through Activating PPAR*γ*

**Figure 4:**
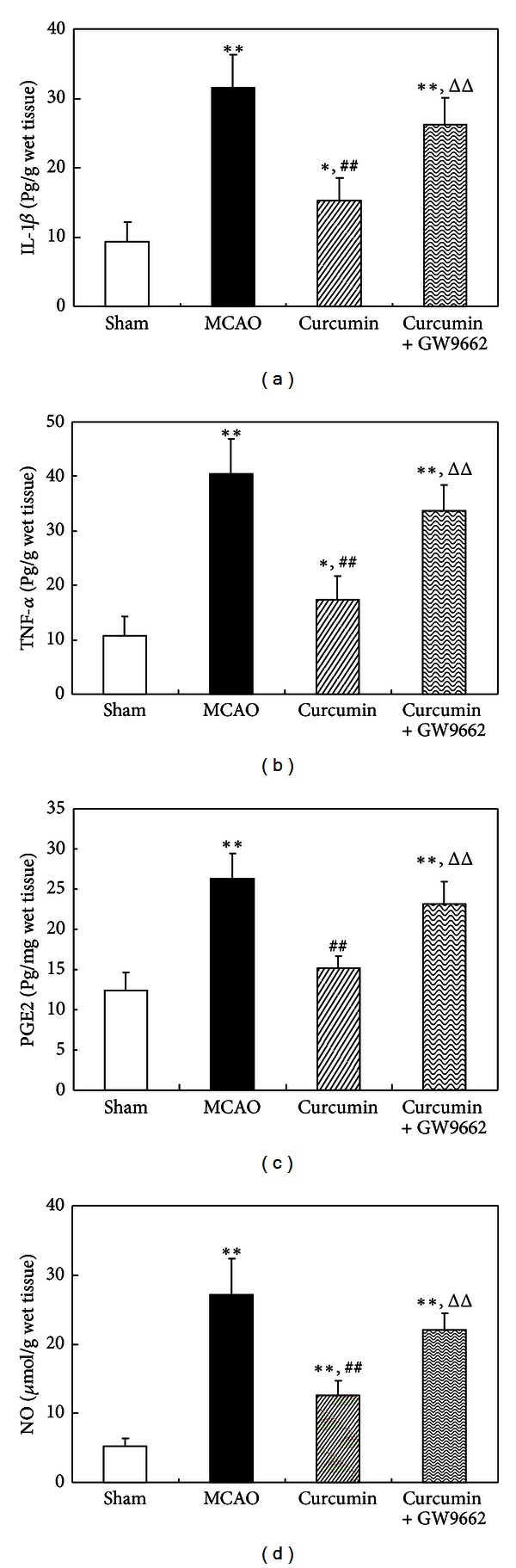
Curcumin inhibited neuroinflammatory response in cerebral ischemia of rat through activating PPAR*γ*. Rats were injected (i.p.) with curcumin and GW9662 for continuous 3 days. One hour after the last injections, the rats were subjected to MCAO for 2 h and reperfused for 24 h. And the cortex was isolated for detection of inflammatory mediators. (a) ELISA assay of IL-1*β*. (b) ELISA assay of TNF-*α*. (c) ELISA assay of PGE2. (d) NO concentration. Data were expressed as mean ± SD with 6 individual rats per group. *P < 0.05, **P < 0.01 versus sham-operated rats, ^##^ *P* < 0.01 versus MCAO rats, ^Δ^ *P* < 0.05, and ^ΔΔ^ *P* < 0.01 versus curcumin-treated rats.

As shown in Figure 4, the productions of IL-1*β*, TNF-*α*, PGE2, and NO significantly increased in rat cortex with cerebral ischemia. Curcumin significantly decreased the levels of IL-1*β*, TNF-*α*, PGE2, and NO, while PPAR*γ* antagonist GW9662 markedly attenuated the inhibitory effect of curcumin on these inflammatory mediators, suggesting that curcumin suppressed inflammatory response in the rat brain through activating PPAR*γ* pathway (Figure 4). 

### 3.5. PPAR*γ* Was Involved in the Suppression of Curcumin on COX-2 and iNOS Expression in Cerebral Ischemia of Rats

**Figure 5:**
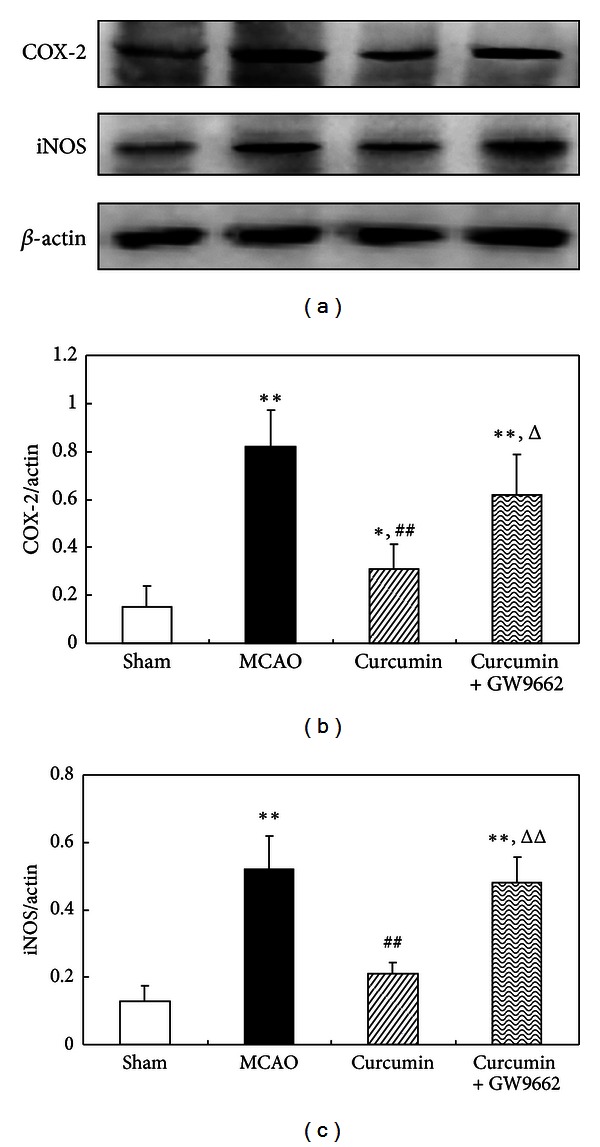
PPAR*γ* was involved in the suppression of curcumin on COX-2 and iNOS expression in cerebral ischemia of rats. Rats were injected (i.p.) with curcumin and GW9662 for continuous 3 days. One hour after the last injections, the rats were subjected to MCAO for 2 h and reperfused for 24 h. And the cortex was isolated for western blot assay. (a) Western blot of COX-2 and iNOS. (b) Statistical of COX-2 expression. (c) Statistical of iNOS expression. Data were expressed as mean ± SD. Western blot images were representative of four independent experiments demonstrating similar results. *P < 0.05, **P < 0.01 versus sham-operated rats, ^##^ *P* < 0.01versus MCAO rats, ^Δ^ *P* < 0.05, and ^ΔΔ^ *P* < 0.01 versus curcumin-treated rats.

Our data showed that the expression of COX-2 increased in cerebral ischemic rats. Treatment of curcumin significantly decreased COX-2 expression. Furthermore, the expression of iNOS significantly increased, and curcumin markedly suppressed iNOS expression in the cortex of rats. The inhibitory effects of curcumin on COX-2 and iNOS expression were attenuated by PPAR*γ* antagonist GW9662 (Figure 5), indicating that the suppression of COX-2 and iNOS by curcumin was PPAR*γ* mediated.

### 3.6. Cerebral Ischemic Injury Was NF-*κ*B-Mediated

**Figure 6:**
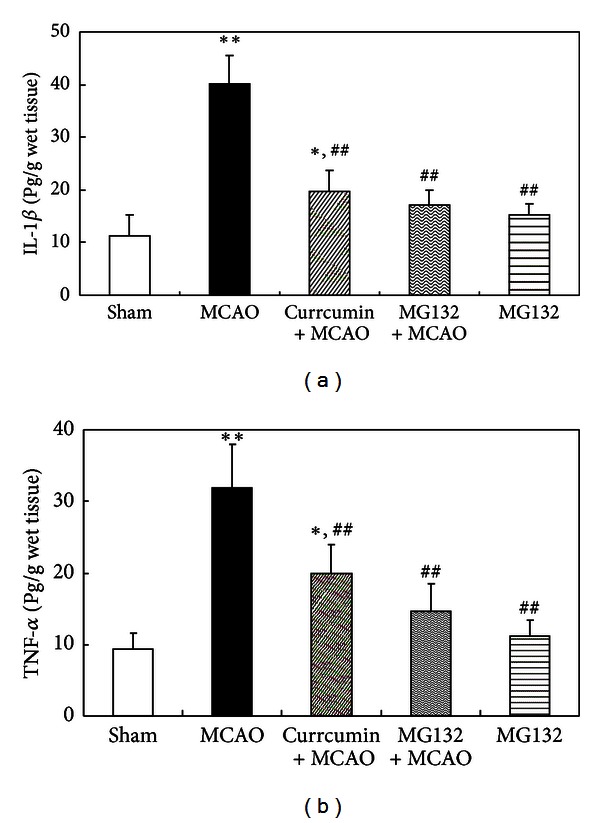
Cerebral ischemic injury was mediated by NF-*κ*B. Rats were injected (i.p.) with curcumin and GW9662 for continuous 3 days. One hour after the last injections, the rats were subjected to MCAO for 2 h and reperfused for 24 h. (a) ELISA assay of IL-1*β*. (b) ELISA assay of TNF-*α*. Data were expressed as mean ± SD with 6 individual rats per group. *P < 0.05, **P < 0.01 versus sham-operated rats, and ^##^ *P* < 0.01 versus MCAO rats.

To further clarify whether the cerebral ischemic injury was mediated by NF-*κ*B, the proteasome inhibitor MG132, which is a well-known NF-*κ*B inhibitor by blocking degradation of I*κ*B-*α*, was employed. MG132 reduced TNF-*α* and IL-1*β* levels 24 h after reperfusion in the cortex of rats, and curcumin decreased the levels of TNF-*α* and IL-1*β* challenged by MCAO (Figure 6). These data indicated that the productions of TNF-*α* and IL-1*β* induced by MCAO were NF-*κ*B mediated and that curcumin decreased TNF-*α* and IL-1*β* productions through the direct inhibition of NF-*κ*B activity. We speculated that curcumin blocked NF-*κ*B activation and subsequently inhibited the transcription of TNF-*α*, IL-1*β*, the target genes of NF-*κ*B, and finally rats were protected from cerebral ischemic injury.

### 3.7. Curcumin-Induced PPAR*γ* Inhibited NF-*κ*B Pathway in Cerebral Ischemia of Rats

**Figure 7:**
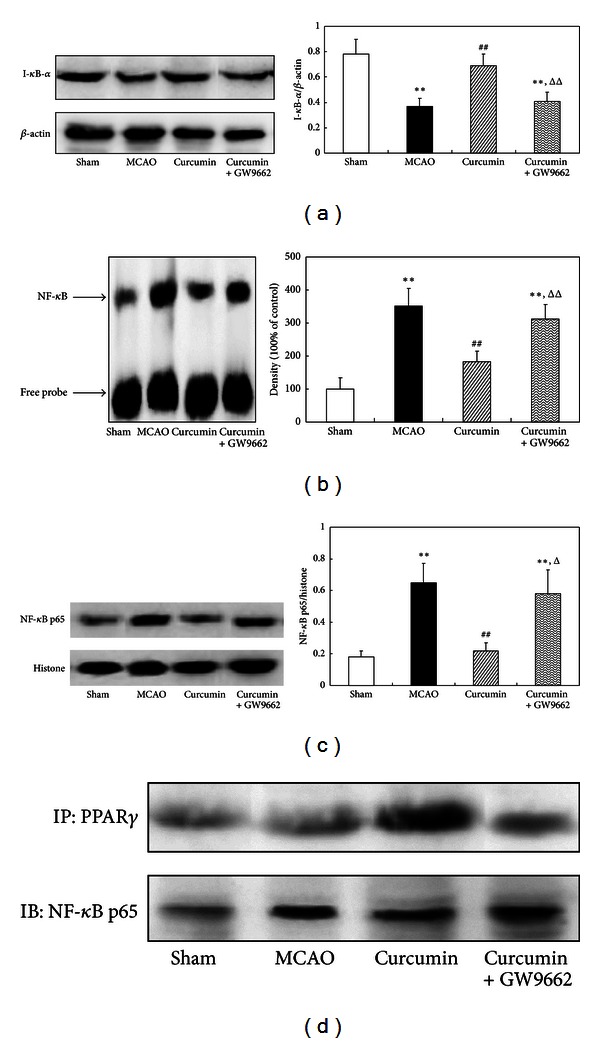
Curcumin-induced PPAR*γ* inhibited NF-*κ*B pathway in cerebral ischemia of rats. Rats were injected (i.p.) with curcumin and GW9662 for continuous 3 days. One hour after the last injections, the rats were subjected to MCAO for 2 h and reperfused for 24 h. (a) Western blot assay of I*κ*B-*α*. (b) EMSA assay of NF-*κ*B-DNA-binding activity. (c) Western blot assay of NF-*κ*B p65 subunit. (d) Co-IP assay of the interaction of PPAR*γ* and NF-*κ*B p65. Data were expressed as mean ± SD. Western blot images were representative of four independent experiments demonstrating similar results. **P < 0.01 versus sham-operated rats, ^##^ *P* < 0.01 versus MCAO rats, ^Δ^ *P* < 0.05, and ^ΔΔ^ *P* < 0.01 versus curcumin-treated rats.

The present results showed that after cerebral ischemia, the degradation of I*κ*B-*α* was observed. Pretreatment of curcumin decreased cerebral ischemia-induced I*κ*B degradation. Coadministration of GW9662 counteracted the inhibitory effect of curcumin on I*κ*B-*α*'s degradation (Figure 7(a)). Thus, this result suggests that curcumin may exert its inhibitory effect on the degradation of I*κ*B-*α* through its activation of PPAR*γ*.

It is becoming increasingly apparent that PPAR*γ* can inhibit NF-*κ*B activity. So we further studied the effect of PPAR*γ* activated by curcumin on NF-*κ*B activation in cerebral ischemic rat model. NF-*κ*B was activated after cerebral ischemia of rat. Pretreatment of curcumin inhibited nuclear translocation of NF-*κ*B p65 subunit and NF-*κ*B-DNA-binding activity. When the PPAR*γ* was inhibited with GW9662, the suppressed nuclear translocation of NF-*κ*B p65 and NF-*κ*B-DNA-binding activity by curcumin was attenuated (Figures 7(b) and 7(c)). Moreover, we also found that PPAR*γ* interacted with NF-*κ*B p65 in curcumin-treated ischemic rats. Blocking of PPAR*γ* with GW9662 reduced this interaction (Figure 7(d)). The results suggested that PPAR*γ* signaling might be involved in the suppression of NF-*κ*B activation in curcumin treated cerebral ischemia rat model.

## 4. Discussion

In this study, using rat cerebral ischemia model, we performed a set of experiments demonstrating that curcumin was a potent PPAR*γ* agonist, which was capable of promoting the survival of neurons, reducing infarct volume, and improving neurobehavioral deficits. In addition, the data also showed the beneficial effects of curcumin against cerebral ischemic injury owing to its suppression of inflammatory response by activating PPAR*γ* signaling, suggesting that modulation of PPAR*γ* activity by curcumin may contribute to improved neuronal survival.

PPAR*γ* is a transcription factor with well-established neuroprotective features [22]. There are increasing evidences demonstrating that pharmacological activation of PPAR*γ* confers to neuroprotection in experimental models of ischemic injury [9, 10], Alzheimer's disease [7], and autoimmune encephalomyelitis [23]. Curcumin has been shown to protect against neuronal injury caused by cerebral ischemia [17, 18]. However, it is unclear whether curcumin protects neuronal injury by activation of PPAR*γ* or not. Present data revealed markedly increases in both expression and activity of PPAR*γ* in the rat cortex by treatment of curcumin, suggesting that curcumin might be a potent agonist of PPAR*γ*. It is also shown that PPAR*γ* agonist is a potent inhibitor of inflammation [24]. A recent study showed that two PPAR*γ* agonists, rosiglitazone and pioglitazone, reduced oxidative stress and inflammatory response induced by ischemia-reperfusion in the rat hippocampus [25]. Consistent with the above studies, our data demonstrated that activation of the PPAR*γ* signaling by curcumin was crucial for all aspects of curcumin-induced protection, as verified by reduction of the curcumin-induced protection through pharmacological inhibition of PPAR*γ* by GW9662. So the activation of PPAR*γ* may serve as an adaptive response to protect neurons against the deleterious effects of cerebral ischemia. Our data, combined with previous studies, suggested that activation of PPAR*γ* by curcumin in neurons after ischemia may represent a prosurvival mechanism against ischemic injury.

Inflammation plays a crucial role in the pathophysiology of cerebral ischemia by producing inflammatory mediators [26], such as IL-1*β*, TNF-*α*, PGE2, NO, COX-2, and iNOS, which are important mediators implicated in the pathology of the ischemic brain. TNF-*α* and IL-1*β* are two well-studied cytokines involved in inflammatory responses after stroke and appeared to aggravate ischemic damage [27]. The therapeutic intervention suppressing the proinflammatory cytokines has been proved to be effective [26, 28]. In the present study, we observed elevated levels of IL-1*β* and TNF-*α* in ischemic brain and successfully demonstrated that curcumin administration prior to ischemia inhibited the level of cytokines in the brain. Moreover, in ischemic brain, inflammation is also attributed by a number of inducible enzymes like COX-2 and iNOS. Therefore, it becomes imperative to evaluate the role of these enzymes in inhibition of ischemic injury by curcumin. COX-2 and iNOS are upregulated in cerebral ischemia and result in the further production of large amounts of PGE2 and NO, which might exacerbate the brain damage [29]. Further more, COX-2-deficient mice [30] and rat knock of iNOS [31] both reduced brain injury induced by MCAO. Present data also demonstrated that COX-2 and iNOS contributed to cerebral ischemia, and curcumin markedly reduced COX-2 and iNOS expression. These results indicated that curcumin is potent anti-inflammatory agent.

 An important role of PPAR*γ* on brain injury is its anti-inflammatory effects [32], which act to silence a broad range of inflammatory genes in microglia, macrophages, and the vasculature [33]. PPAR*γ* deficiency increases susceptibility to brain damage after cerebral ischemia [34], while activation of PPAR*γ* may attenuate ischemic injury through suppressing neuroinflammation [13]. PPAR*γ* agonists were shown to suppress cytokine evoked neuronal COX-2 and iNOS expression, and thereby preventing PGE2 and NO mediated cell death of neurons. Studies have shown that pioglitazone expressing PPAR*γ* suppressed COX-2 expression in rat cortical neurons [25] and PPAR*γ*-agonist WY14643 suppressed oxidative stress and expression of iNOS in ischemia-reperfusion injury in the rat hippocampus [26]. Correlated with the above studies, our data showed that curcumin markedly suppressed inflammatory mediators, such as IL-1*β*, TNF-*α*, NO, PGE2, COX-2, and iNOS levels in response to cerebral ischemia. These inhibitory effects of curcumin were reversed after cotreatment with GW9662, a selective antagonist of PPAR*γ*, clearly demonstrating a PPAR*γ*-dependent mechanism in the neuroprotection of curcumin. 

NF-*κ*B is a ubiquitous transcription factor that regulates a number of genes involved in inflammation and immune response, which is normally sequestered in the cytoplasm where it associates with a family of inhibitory proteins known as I*κ*B. Activation of NF-*κ*B after cerebral ischemia induced expression of proinflammatory genes including TNF-*α*, IL-1*β*, and COX-2. Inhibiting the activation of NF-*κ*B leads to reduced infarcts in the acute stage of cerebral ischemia [35]. Previous researches and our results have shown that, as a pivot regulator of inflammation, NF-*κ*B is activated and contributes to ischemia-induced brain injury [36]. Recently, the inhibitory effects of PPAR*γ* on NF-*κ*B activation are increasingly being demonstrated in different cell systems. Activation of NF-*κ*B is critically regulated at multiple steps. As to PPAR*γ*, it suppresses proinflammatory gene expression at the transcriptional level through inhibiting NF-*κ*B activation [32]. In the present study, PPAR*γ* physically interacted with NF-*κ*B p65 subunit, blocked NF-*κ*B activation, and finally inhibited the dependent gene expression. These results indicated that activation of PPAR*γ* was involved in the inhibition of NF-*κ*B pathway in curcumin-treated cerebral ischemia of rats.

The major focus of the present work concentrated on PPAR*γ* in protecting against cerebral ischemic injury. Our study not only provides the first evidence that PPAR*γ* induced by curcumin may play critical roles in protecting against brain injury through suppression inflammatory response but also highlights the potential of curcumin as a therapeutic agent against cerebral ischemia.

## References

[1] Kaushal V, Schlichter LC (2008). Mechanisms of microglia-mediated neurotoxicity in a new model of the stroke penumbra. *Journal of Neuroscience*.

[2] Bi X, Yan B, Fang S (2009). Quetiapine regulates neurogenesis in ischemic mice by inhibiting NF-*κ*B p65/p50 expression. *Neurological Research*.

[3] Berger J, Moller DE (2002). The mechanisms of action of PPARs. *Annual Review of Medicine*.

[4] Burns KA, Vanden Heuvel JP (2007). Modulation of PPAR activity via phosphorylation. *Biochimica et Biophysica Acta*.

[5] Bernardo A, Ajmone-Cat MA, Gasparini L, Ongini E, Minghetti L (2005). Nuclear receptor peroxisome proliferator-activated receptor-*γ* is activated in rat microglial cells by the anti-inflammatory drug HCT1026, a derivative of flurbiprofen. *Journal of Neurochemistry*.

[6] Cimini A, Benedetti E, Cristiano L (2005). Expression of peroxisome proliferator-activated receptors (PPARs) and retinoic acid receptors (RXRs) in rat cortical neurons. *Neuroscience*.

[7] Fakhfouri G, Ahmadiani A, Rahimian R, Grolla AA, Moradi F, Haeri A (2012). WIN55212-2 attenuates amyloid-beta-induced neuroinflammation in rats through activation of cannabinoid receptors and PPAR-*γ* pathway. *Neuropharmacology*.

[8] Garrido-Gil P, Joglar B, Rodriguez-Perez AI, Guerra MJ, Labandeira-Garcia JL (2012). Involvement of PPAR-*γ* in the neuroprotective and anti-inflammatory effects of angiotensin type 1 receptor inhibition: effects of the receptor antagonist telmisartan and receptor deletion in a mouse MPTP model of Parkinson's disease. *Journal of Neuroinflammation*.

[9] Gillespie W, Tyagi N, Tyagi SC (2011). Role of PPAR*γ*, a nuclear hormone receptor in neuroprotection. *Indian Journal of Biochemistry and Biophysics*.

[10] Abdelrahman M, Sivarajah A, Thiemermann C (2005). Beneficial effects of PPAR-*γ* ligands in ischemia-reperfusion injury, inflammation and shock. *Cardiovascular Research*.

[11] Tureyen K, Kapadia R, Bowen KK (2007). Peroxisome proliferator-activated receptor-*γ* agonists induce neuroprotection following transient focal ischemia in normotensive, normoglycemic as well as hypertensive and type-2 diabetic rodents. *Journal of Neurochemistry*.

[12] Victor NA, Wanderi EW, Gamboa J (2006). Altered PPAR*γ* expression and activation after transient focal ischemia in rats. *European Journal of Neuroscience*.

[13] Sundararajan S, Gamboa JL, Victor NA, Wanderi EW, Lust WD, Landreth GE (2005). Peroxisome proliferator-activated receptor-*γ* ligands reduce inflammation and infarction size in transient focal ischemia. *Neuroscience*.

[14] Feinstein DL, Spagnolo A, Akar C (2005). Receptor-independent actions of PPAR thiazolidinedione agonists: is mitochondrial function the key?. *Biochemical Pharmacology*.

[15] Barinaga M (1998). Stroke-damaged neurons may commit cellular suicide. *Science*.

[16] Dutta S, Padhye S, Priyadarsini KI, Newton C (2005). Antioxidant and antiproliferative activity of curcumin semicarbazone. *Bioorganic and Medicinal Chemistry Letters*.

[17] Lim CS, Jin DQ, Mok H (2005). Antioxidant and antiinflammatory activities of xanthorrhizol in hippocampal neurons and primary cultured microglia. *Journal of Neuroscience Research*.

[18] Zhao J, Yu S, Zheng W (2010). Curcumin improves outcomes and attenuates focal cerebral ischemic injury via antiapoptotic mechanisms in rats. *Neurochemical Research*.

[19] Liu ZJ, Bao XQ, Jia SH (2011). Constructing the screening cell model of PPAR*γ* and to validate whether the curcumin is the natural agonist of PPAR*γ*. *Journal of Apoplexy and Nervous Diseases*.

[20] Swanson RA, Morton MT, Tsao-Wu G, Savalos RA, Davidson C, Sharp FR (1990). A semiautomated method for measuring brain infarct volume. *Journal of Cerebral Blood Flow and Metabolism*.

[21] Longa EZ, Weinstein PR, Carlson S, Cummins R (1989). Reversible middle cerebral artery occlusion without craniectomy in rats. *Stroke*.

[22] Singh S, Khar A (2006). Biological effects of curcumin and its role in cancer chemoprevention and therapy. *Anti-Cancer Agents in Medicinal Chemistry*.

[23] Hucke S, Floßdorf J, Grützke B (2012). Licensing of myeloid cells promotes central nervous system autoimmunity and is controlled by peroxisome proliferator-activated receptor ?. *Brain*.

[24] Luna-Medina R, Cortes-Canteli M, Alonso M, Santos A, Martínez A, Perez-Castillo A (2005). Regulation of inflammatory response in neural cells in vitro by thiadiazolidinones derivatives through peroxisome proliferator-activated receptor *γ* activation. *Journal of Biological Chemistry*.

[25] Collino M, Aragno M, Mastrocola R (2006). Modulation of the oxidative stress and inflammatory response by PPAR-*γ* agonists in the hippocampus of rats exposed to cerebral ischemia/reperfusion. *European Journal of Pharmacology*.

[26] Sieber MW, Claus RA, Witte OW, Frahm C (2011). Attenuated inflammatory response in aged mice brains following stroke. *PLoS ONE*.

[27] Chan SJ, Wong WSF, Wong PTH, Bian JS (2010). Neuroprotective effects of andrographolide in a rat model of permanent cerebral ischaemia. *British Journal of Pharmacology*.

[28] Yasuda Y, Shimoda T, Uno K (2011). Temporal and sequential changes of glial cells and cytokine expression during neuronal degeneration after transient global ischemia in rats. *Journal of Neuroinflammation*.

[29] Krakauer T (2004). Molecular therapeutic targets in inflammation: cyclooxygenase and NF-*κ*B. *Current Drug Targets*.

[30] Sasaki T, Kitagawa K, Yamagata K (2004). Amelioration of hippocampal neuronal damage after transient forebrain ischemia in cyclooxygenase-2-deficient mice. *Journal of Cerebral Blood Flow and Metabolism*.

[31] Atochin DN, Clark J, Demchenko IT, Moskowitz MA, Huang PL (2003). Rapid cerebral ischemic preconditioning in mice deficient in endothelial and neuronal nitric oxide synthases. *Stroke*.

[32] Daynes RA, Jones DC (2002). Emerging roles of PPARs in inflammation and immunity. *Nature Reviews Immunology*.

[33] McKay LI, Cidlowski JA (1999). Molecular control of immune/inflammatory responses: interactions between nuclear factor-*κ*B and steroid receptor-signaling pathways. *Endocrine Reviews*.

[34] Zhao X, Strong R, Zhang J (2009). Neuronal PPAR*γ* deficiency increases susceptibility to brain damage after cerebral ischemia. *Journal of Neuroscience*.

[35] Ridder DA, Schwaninger M (2009). NF-*κ*B signaling in cerebral ischemia. *Neuroscience*.

[36] Zhang HL, Xu M, Wei C (2011). Neuroprotective effects of pioglitazone in a rat model of permanent focal cerebral ischemia are associated with peroxisome proliferator-activated receptor gamma-mediated suppression of nuclear factor-*κ*B signaling pathway. *Neuroscience*.

